# Elucidating the Role of Graphene Oxide Surface Architecture and Properties in Loess Soil Remediation Efficacy

**DOI:** 10.3390/nano15141098

**Published:** 2025-07-15

**Authors:** Zirui Wang, Haotian Lu, Zhigang Li, Yuwei Wu, Junping Ren

**Affiliations:** 1 School of Tropical Agriculture and Forestry, Hainan University, Haikou 570228, China; 20223007393@hainanu.edu.cn (Z.W.);; 2 College of Civil Engineering and Mechanics, Lanzhou University, Lanzhou 730000, China; 3 School of Civil and Architectural Engineering, Hainan University, Haikou 570228, China

**Keywords:** graphene oxide, Lanzhou loess, disintegration experiment, soil-water characteristic curve

## Abstract

Loess Plateau is the region with the most concentrated loess distribution and the deepest loess soil layer in the world, and it is facing serious problems of soil erosion and ecological degradation. The nano carbon modification of soil surface properties is a novel strategy for soil improvement and enhancing the soil’s capacity to sequester carbon, which has been extensively researched. However, the mechanisms underlying the influence of carbon surface structure on the efficacy of loess soil remediation remain unclear. Herein, graphene oxide (GO) with a unique two-dimensional structure and adjustable surface properties was optimized as a model carbon filler to investigate the modification effect on loess. As a result, the addition amount of 0.03% GO significantly reduced the disintegration amount of loess, but, if inhibited for a long time, the disintegration effect would weaken. The highly reduced GO can delay the loess disintegration rate due to its enhanced hydrophobicity, but the inhibitory effect fails over a long period of time. After adjusting the reduce degree with a 50% SA (sodium ascorbate), the water-holding capacity of the modified soil in the high suction range is enhanced. This study reveals the synergistic mechanism of the sheet structure and surface properties of GO on the water stability of loess, providing a reference for the prevention and control of soil erosion and ecological restoration in the Loess Plateau.

## 1. Introduction

The Loess Plateau spans the north-central part of China, covering an area of about 640,000 square kilometers. It is the region with the most concentrated loess distribution and the deepest soil layer in the world [1]. However, this area is also one of the most ecologically fragile regions with the most severe soil erosion in the world. According to statistics, the Loess Plateau has an average annual soil erosion amount of 5000 to 15,000 tons per square kilometer, accounting for more than 40 percent of the total soil erosion in the country [2]. Severe soil erosion not only leads to land degradation and the reduction of arable land but also triggers secondary disasters such as the siltation of rivers and frequent floods, seriously threatening regional food security and ecological security. The core cause lies in the special physicochemical properties of the loess: the loess is mainly composed of fine particles, with a porosity of up to 40 to 50 percent but with low organic matter content and insufficient clay content, resulting in a loose structure and poor water and nutrient retention capacity [3]. Under climate conditions of concentrated and intense rainfall, the topsoil is prone to disintegration and erosion under runoff, resulting in the landform feature of “thousands of gullies and ravines”. Therefore, how to enhance the structural stability and water regulation capacity of loess through soil improvement techniques has become a key issue for ecological restoration and sustainable development in this region.

Traditional soil improvement techniques mainly rely on the application of organic fertilizers, chemical agents such as lime, cement, or vegetation covering, etc. However, these methods have significant limitations in practical applications: the improvement cycle of organic fertilizers is long, and they are prone to being eroded by runoff [4]. Chemical agents may destroy the activity of soil microorganisms and cause secondary salinization [5]. Vegetation restoration is limited by the poor water and fertilizer conditions of loess [6]. In recent years, nanomaterials have provided new ideas for soil improvement due to their unique surface effects, small size effects, and high reactivity. For example, nano-clay can change the stability of soil aggregates by blocking pores [7]. Carbon nanotubes can enhance the shear strength of soil through bridging [8]. However, most nanomaterials have problems such as high cost and uncertain environmental risks, which limit their large-scale application. Persistent pollutants such as trace metals and quasi-metals accumulate in the urban environment, which may lead to urban runoff pollution [9]. Against this background, Graphene Oxide (GO), as a new type of carbon-based nanomaterial, despite having disadvantages such as easy aggregation and difficulty in separation [10], demonstrates significant application potential in soil improvement, agricultural production, and pollutant treatment [11,12,13].

GO is a two-dimensional nanosheet material formed by the oxidation and exfoliation of graphene. Its surface is rich in oxygen-containing functional groups such as hydroxyl, epoxy, and carboxyl groups, and the layers are stacked through hydrogen bonds and van der Waals forces [14]. This unique structure endows GO with multiple advantages: Firstly, its high specific surface area and abundant functional groups endow it with extremely strong adsorption capacity, which can effectively fix heavy metal ions [15] and nutrient elements [16]. Secondly, the layered structure of GO can fill soil pores and enhance the intergranular binding force through electrostatic interaction with clay particles [17]. In addition, the oxidation degree of GO can be regulated through chemical reduction, thereby flexibly changing its hydrophilicity/hydrophobicity and dispersibility [18]. These characteristics enable GO to demonstrate unique potential in improving soil structure, regulating water transport, and enhancing erosion resistance. For instance, Ren et al. (2016) [12] found that adding 0.01% GO could increase the shear strength of clay by 30%. Furthermore, the antibacterial function of GO [19] and the sustained-release effect of pesticides [20] have further expanded its application scenarios in agricultural ecological restoration. However, it is important to highlight that GO exhibits persistence and mobility within environmental systems. It can readily form stable colloidal suspensions in aquatic environments and may induce toxic effects on aquatic organisms and terrestrial plants via mechanisms such as oxidative stress. Consequently, the ecological risks associated with prolonged low-dose exposure warrant careful consideration [21,22].

Although the soil improvement potential of GO has been preliminarily verified, there is still a significant gap in the research on its application in loess. Firstly, the oxidation degree of GO directly affects the types and distribution of its surface functional groups, thereby determining its interaction mode with soil particles. For example, high-oxidation-degree GO (oxygen content > 40%) may enhance soil water-holding capacity through hydrogen bonds because it is rich in hydrophilic groups. However, excessive hydrophilic groups may also cause an excessive swelling of GO sheets, which instead damages the soil structure [23]. However, low-oxidation-degree GO (oxygen content < 10%) may inhibit water penetration by forming a waterproof barrier due to enhanced hydrophobicity, but excessive accumulation will reduce the uniformity of dispersion [24]. However, most of the existing studies focus on the influence of a single oxidation degree of GO, lacking systematic comparative analysis of oxidation gradients, resulting in the “oxidation degree–functional effect” relationship of GO not being clear yet. Secondly, the microscopic interaction between GO and loess has not been fully revealed. The mineral composition of loess, which is mainly composed of fine particles and lacking in clay particles, may limit the adsorption sites of GO, and its high porosity may lead to the migration or aggregation of GO sheets [25] Furthermore, the mechanism by which the addition amount of GO affects the soil-water characteristic curve (SWCC) remains unclear: A small amount of GO may enhance the water-holding capacity by filling pores, while a high addition amount may form a discontinuous barrier due to layer blockage, thereby reducing water availability [26] The existence of these problems leads to the lack of theoretical guidance for the optimal application of GO in loess improvement.

In response to the above deficiencies, this study took the typical loess of Lanzhou as the research object. By regulating the reduction degree (0%, 10%, 50%, 100%) and addition amount (0.003–0.03%) of GO, a series of composite soil specimens were prepared, and multi-scale experimental studies were carried out: Combining macroscopic disintegration experiments, SWCC tests, and microscopic analysis by scanning electron microscopy (SEM), the influence mechanism of GO on the water-holding capacity and stability of loess was systematically explored. This study not only provides a basis for revealing the GO–loess interaction but also lays a foundation for the application of GO in ecological engineering in arid and semi-arid areas.

## 2. Experimental Materials and Details

### 2.1. Materials Preparation

The test soil is a typical loess collected from natural ground surface. The sampling site (36°10′N, 103°47′E) is located at Jiuzhoutai, Lanzhou City, Gansu Province, China. It is among the thickest loess deposits on Earth, with a thickness of up to 335 m [1]. The collected soil specimens were air-dried, crushed, and sieved through a 2 mm standard sieve. According to the Unified Soil Classification System (USCS) (ASTM D2487-2011, West Conshohocken, USA), loess is classified as silty clay. The GO used in this study is a major derivative of graphene. Although GO retains the layered structure of graphene, it is decorated with numerous oxygen-containing functional groups on its surface. The introduction of these functional groups makes the graphene structure highly complex, requiring microscopic techniques for characterization.

### 2.2. Macroscopic Testing

#### 2.2.1. Physical Properties of the Specimen

In the redox reaction between GO and sodium ascorbate (SA), SA acts as a strong reducing agent, transferring electrons to the oxygen-containing functional groups of GO. When GO is reduced, some oxygen groups are removed, and the conjugated structure of the carbon skeleton is gradually restored [24]. SA was reacted with GO to prepare four distinct reduction gradients—0%, 10%, 50%, and 100%–and then the four GO with different reduction degrees were mixed with loess to form specimens. The dry weight ratios of GO to loess were 0.03%, 0.01%, 0.005%, and 0.003%, respectively.

**Figure 1:**
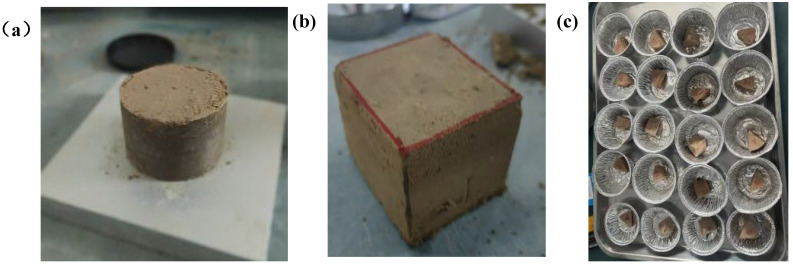
(**a**) The compacted GO–loess sample, (**b**) the cubic sample used for the disintegration experiment, and (**c**) the small sample block used for the SWCC test.

The specific process of preparing GO–loess specimens is as follows: The collected soil specimens that have passed through a 2 mm standard sieve are placed in a drying oven and dried at 105 °C for 24 h. The dried soil was taken out and mixed with GO to prepare a sample with a target moisture content of 14% (by weight) and a dry density of 1.45 g/cm^3^. A thin layer of Vaseline was applied to the inner wall of the soil cutting ring used in the test to reduce the friction on the side wall. The cutting edge was placed downward on the soil sample. A scraper was used to evenly spread the soil sample into the cylinder to avoid particle segregation and keep the thickness of each loose layer consistent. It was loaded in two layers, with each layer’s soil volume accounting for approximately half of the total height. After each layer was compacted, a scraper was used to “roughen” the surface (loosening about 2 mm of the surface layer) to ensure interlayer bonding. After the compaction was completed, the sample was slowly pushed out to avoid its breakage. The specific experimental materials are shown in Figure 1.

#### 2.2.2. Lanzhou Loess Disintegration Test

The soil disintegration test was conducted in accordance with the “Geotechnical Test Procedures” (GB/T 50123-2019, Beijing, China) for disintegration testing. The main procedures for conducting this test were as follows: (a) A certain amount of GO solution was completely mixed with loess to achieve a target water content of 14% (by weight). The mixture was compacted in a standard steel ring. (b) After compaction, the height of the sample was 5.2 cm, and the diameter was 7.0 cm. It was then cut into cubes with an edge length of 5 cm using a soil scraper. (c) Subsequently, the sample was placed in the center of the mesh plate, which was hung under the float. Then, the neck end of the float was held, and the sample quickly immersed in the water cylinder. (d) The stopwatch was started, and the instantaneous stable reading and the start time of the scale at the water level of the float at the beginning were recorded. The scale readings were then recorded at intervals of 1 min, 3 min, 5 min, 8 min, 10 min, 13 min, 15 min, etc. The test was over when the sample had completely passed through the grid and fallen.

**Figure 2:**
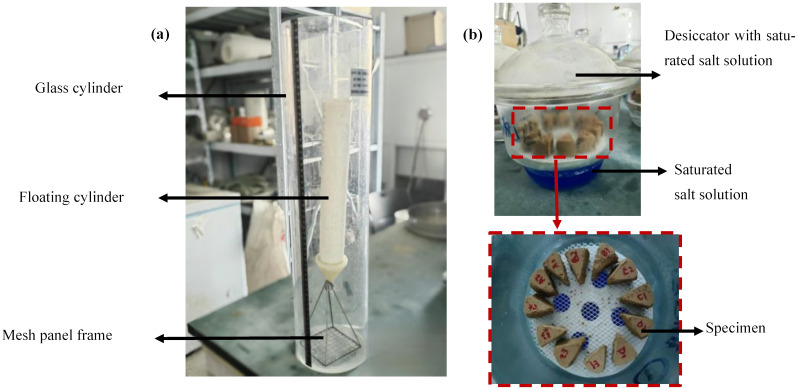
(**a**) Disintegration tester and (**b**) vapor equilibrium method for SWCC measurement of specimens.

In the experiment, four redox gradients of 0%, 10%, 50%, and 100% were respectively set up. The ratio of GO to the dry mass of loess was 0.03%. Four different dry mass ratios of graphene oxide–clay were set up, specifically 0.03%, 0.01%, 0.005%, and 0.003%, with the reduction degree of GO at 0% (pure GO), and the disintegration amounts at different time nodes were determined. Each group of treatments was repeated three times. The experimental equipment is detailed in Figure 2a.

#### 2.2.3. Lanzhou Loess SWCC Test

The soil water characteristic curve (SWCC) represents the relationship between soil water suction and soil moisture content, indicating the relationship between the energy and quantity of soil water. It is a curve used to reflect the basic characteristics of soil moisture in the study of soil moisture retention and movement. The measurement of SWCC was carried out using compacted specimens. A certain amount of GO and loess was mixed completely and placed in a sealed bag to achieve a target moisture content of 14% (by weight). The mixture was compacted in a standard steel ring. After compaction, the height of the specimen was 2 cm, and the diameter was 6.18 cm. Subsequently, the specimens were saturated using a vacuum saturation device, and the mass of the saturated specimens was recorded.

This study adopted the vapor equilibrium method with a high suction range (3000–3,000,000). The principle of the vapor equilibrium method is that, under constant temperature conditions, the relative humidity above the saturated salt solution is fixed. According to the Kelvin equation, this corresponds to a constant suction force. In this study, we used eight different saturated salt solutions. The saturated specimens were cut into small pieces and placed in a desiccator with eight different solutions. The entire device was maintained at approximately 20 °C for 4 weeks to reach equilibrium, and then the moisture content and suction of the sample were determined. Each group of treatments was repeated three times. See Figure 2b for details.

### 2.3. Microscopic Testing

The interlayer structure of GO was characterized by XRD. Through the measurement of diffraction patterns, the characteristic peaks corresponding to graphene oxide were identified. The existence and changes of functional groups in GO were investigated using FTIR. By analyzing the infrared absorption spectra, specific characteristic absorption peaks associated with these functional groups could be determined. Furthermore, by examining the XPS of samples with varying degrees of oxidation, a deeper understanding of the surface chemical properties of GO and their correlation with performance was achieved.

Microscopic analysis was carried out using scanning electron microscopy (SEM) to further study the influence of GO on the properties of loess in Lanzhou. To achieve this purpose, compacted specimens were prepared following the same procedure as for SWCC measurement. Furthermore, in order to preserve the original structure of BAS specimens, these small specimens were freeze-dried before being tested under a microscope. Through scanning electron microscopy analysis, the visible morphology and microstructure of the specimens and the interaction between GO and soil were derived.

A 50 μL flat-tipped micro-syringe (needle tip outer diameter 0.7 mm, solid PTFE plunger) was used. The flat-tipped needle was placed vertically above the substrate, and the droplet was slowly injected. The needle was immediately withdrawn after the droplet was released to ensure that the droplet shape was only affected by surface tension. The static images of the droplets were captured using an optical contact angle meter to verify the hydrophilicity and hydrophobicity of GO with different oxidation degrees through contact angle tests.

## 3. Experimental Results and Discussion

### 3.1. Influence on the Disintegration of Lanzhou Loess

Disintegration rate is an important indicator used to describe the disintegration speed of soil specimens, reflecting the water resistance of the soil. The disintegration rate is not an instantaneous value but an average value within a given time period, with the unit of %/min. The calculation formula for disintegration amount (%) is:

(1) $$A1=\frac{R_{t}-R_{0}}{100-R_{0}}\times 100$$

In the formula: *A_1_*—The disintegration amount of the sample at time t (%); *R_t_*—The scale reading at the water level of the float at time t; *R_0_*—The instantaneous stable reading of the scale at the water level of the float at the beginning of the test.

**Figure 3:**
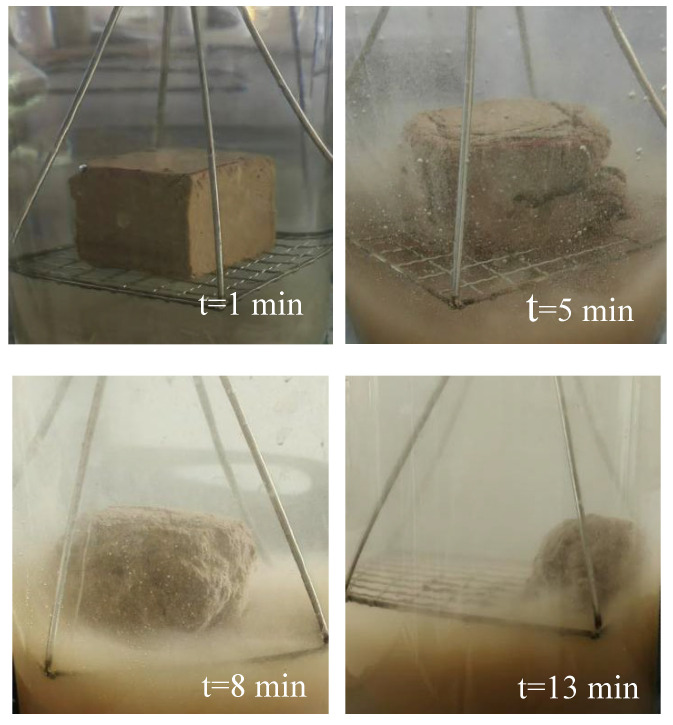
Specimens in disintegration experiments.

After the soil sample is immersed in water, the surface soil particles would rapidly diffuse, immediately generating a large number of bubbles. Cracks would start to appear at the boundary of the soil sample, and the soil at the edge would collapse along the cracks to form broken particles, making the clear water turbid. It was observed that the sample began to disintegrate from the bottom first, and the disintegration rate gradually increased. The area expanded and extended to the top, and gaps began to appear on the top surface. In the middle and later stages, the bubble diameters were small but dense, occasionally accompanied by large bubbles, and the disintegration rate gradually slowed down, as shown in Figure 3.

**Figure 4:**
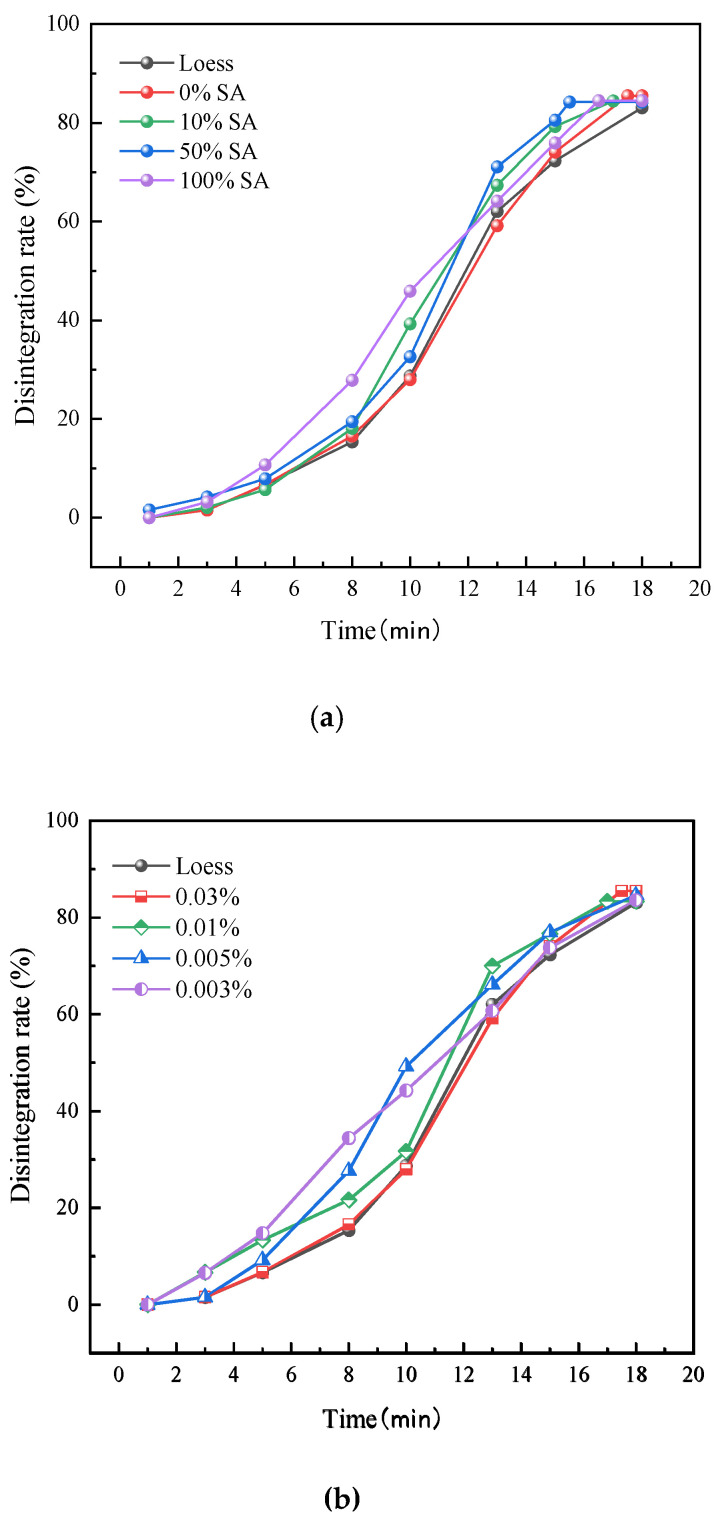
The disintegration rate and time of (**a**) different restoration degrees specimens and (**b**) different dosage levels specimens.

Figure 4 summarizes the disintegration rate and time of specimens with different GO reduction degrees and dosage levels. Figure 4a shows that, with the increased SA dosage, the reduction degree of GO improves, resulting in enhanced hydrophobicity. The hydrophilicity of GO mainly comes from the oxidizing groups on its surface (e.g., hydroxyl, carboxyl, etc.). The reduction of these groups leads to a decrease in the dispersibility of GO in water, an increase in hydrophobicity, and an intensification of flake stacking, which may result in a reduction in the effective specific surface area of GO, affecting its combination with soil and causing an increase in disintegration [23]. The highly-reduced-degree GO is highly hydrophobic, forming a dense lamellar structure that hinders the rapid infiltration of water into the gaps between soil particles and delays the disintegration process. However, the stacking of flakes may lead to a decrease in dispersion, forming discontinuous local hydrophobic barriers, resulting in large fluctuations in the disintegration amount in the early stage. The low reduction degree of GO still retains a large number of hydrophilic groups, has a strong binding ability with water, accelerates water penetration, and has a relatively high disintegration amount. At 18 min, the specimens had basically completed disintegration. The disintegration amounts of the specimens with 0% SA, 10% SA, 50% SA, and 100% SA were 85.49%, 84.45%, 84.21%, and 84.5%, respectively. Thus, the disintegration amount of the sample with 50% SA (medium degree of reduction) was the lowest among all groups and was close to that of natural loess. Moreover, the 50% reduction group completed disintegration first, and the time taken was significantly shorter compared to other groups, indicating that the optimal disintegration inhibition effect was achieved with a moderate reduction of GO.

Different dosages of GO have a significant impact on the disintegration of loess, as shown in Figure 4b. As the concentration of GO increases, the disintegration amount decreases. The disintegration amount of the sample with a 0.03% concentration is overall lower than that of other concentrations. During the initial and stable stages, its disintegration amount approaches that of natural loess. This indicates that a high concentration of GO can effectively inhibit the disintegration of loess, but the disintegration rate reaches 74.07% after 15 min, which is close to the disintegration rate of 73.77% in the low-concentration group, reflecting the failure of the inhibition effect. Thus, the initial inhibitory effect of a high concentration of GO is significant, but the long-term hydration expansion fails; the low concentration coverage is insufficient, and the inhibitory effect on disintegration is limited.

### 3.2. Impact on SWCC of Lanzhou Loess

The sample achieves a specific total suction force by balancing with the humidity environment. By determining the moisture content of the specimens at equilibrium under different saturated salt solutions, the relationship between moisture content and total suction force was established. The relationship between suction and relative humidity is expressed by the Kelvin equation. At 20 °C, the Kelvin equation is:

(2) S=−135022ln(RH)

where: *S*—suction force (kPa); *RH*—Relative humidity.

An elevation in the oxidation degree is associated with a decline in the soil’s water-holding capacity. In the high-suction zone, the water content of the highly oxidized group rises, which indicates its enhanced hydrophilicity. In the high-suction segment, the water-holding capacities of GO with varying oxidation-reduction gradients exhibit notable discrepancies. Specifically, the sample with 0% SA demonstrates the lowest water retention capacity. The surface of the unreduced GO is abundant in oxygen-containing functional groups, creating hydrophilic micro-regions [27]. However, this results in a limited water-holding ability. For the sample with 10% SA, its water-holding capacity increases slightly. During the reduction process, the partial removal of oxygen groups causes a decrease in its hydrophilicity. The sample with 50% SA displays a relatively better water-holding capacity, maintaining a consistently high water content. As for the 100% SA sample, its water-holding capacity recovers. After complete reduction, the GO layers restack to form a dense structure, which in turn reduces the water-holding ability.

**Figure 5:**
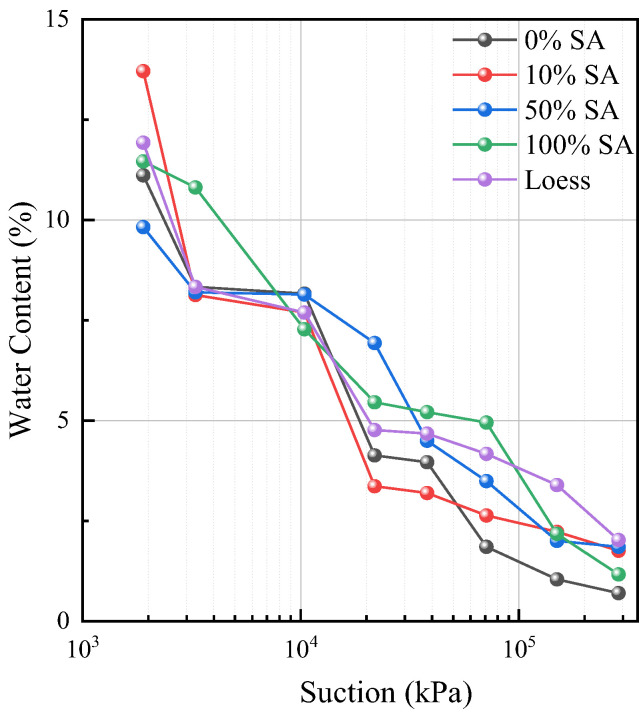
The SWCC of BAS measured by the vapor equilibrium method.

A decrease in oxidation degree (an increase in SA proportion) leads to a reduction in oxygen functional groups and an enhancement in the hydrophobicity of GO. The experiment shows that moderate reduction enhances soil water retention ability while excessive reduction has a negative effect. The relevant data are presented in Figure 5.

### 3.3. Influence on the Microstructure of Lanzhou Loess

**Figure 6:**
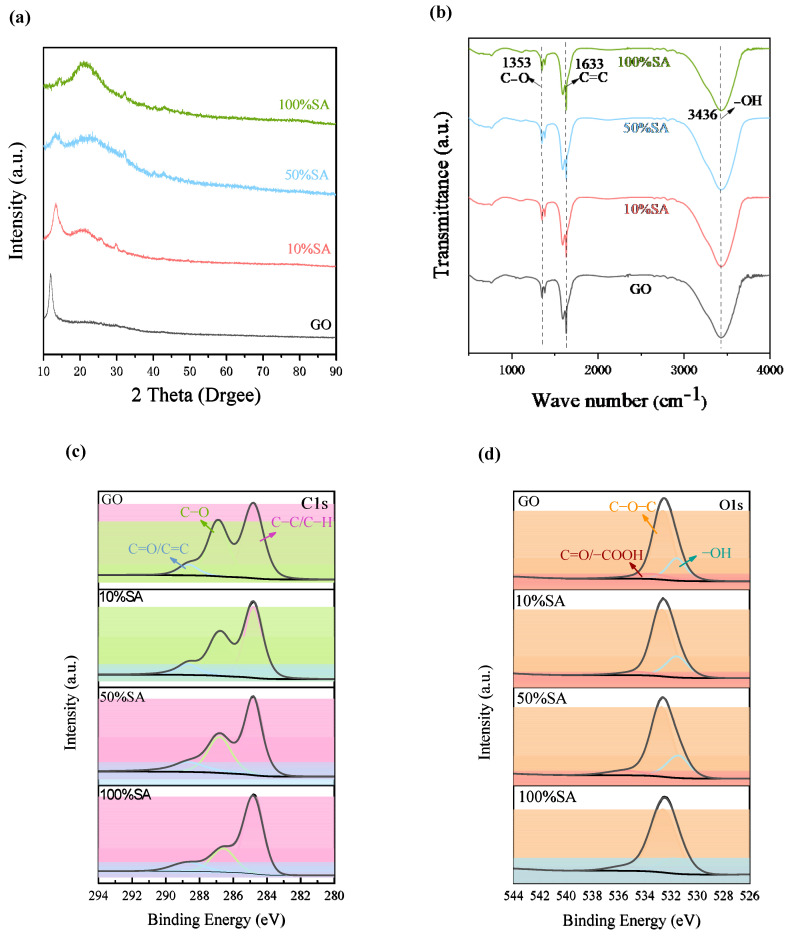
(**a**) XRD patterns of GO with varying degrees of reduction, (**b**) FTIR spectrum of GO with varying degrees of reduction, and (**c**) C1s and (**d**) O1s XPS spectra.

Based on the analysis of the microstructure of Lanzhou loess and the typical structural features of GO, the following observations were made:

Figure 6a is the X-ray diffraction pattern of the GO with varying degrees of reduction. The peak at 13° is the characteristic peak of (002) in GO, indicating a successful synthesis of GO. As the sodium ascorbate ratio increases, the characteristic peak at (002) in GO gradually widens and shifts towards higher angles, demonstrating that the GO was effectively reduced by SA and gradually stacked.

According to Figure 6b, the functional groups corresponding to each wave number are as follows: The peak intensities at 3436 cm^−1^ and 1353 cm^−1^ in the 0% SA group were significantly high, indicating the abundance of oxygen-containing groups such as hydroxyl and epoxy groups. 1633 cm^−1^ might be dominated by C=O, reflecting the presence of carboxylic acid groups; 50% SA: The intensity of the O-H peak decreases, indicating that the hydroxyl group is partially reduced. The peak at 1633 cm^−1^ shifts towards the C=C (sp^2^ structure), indicating a partial recovery of graphene. The peaks of 1353 cm^−1^ and 1000 cm^−1^ weakened, and the epoxy groups and C-O bonds decreased. 100%SA: The O-H peak is further weakened or even nearly disappears, reflecting that the hydroxyl group is almost completely removed; the peak intensity was enhanced and sharpened at 1633 cm^−1^, indicating that the C=C (sp^2^ structure) was dominant and the carboxylic acid group was reduced. The peaks of 1353 cm^−1^ and 1000 cm^−1^ are almost invisible, indicating a significant reduction in the epoxy group and C-O bond. 1633 cm^−1^ changes from a wide peak (C=O) to a sharp peak (C=C), directly reflecting the reduction effect; the attenuation of the 3436 cm^−1^ peak indicates a decrease in hydrophilicity and an increase in hydrophobicity. The oxygen-containing groups of unreduced GO are abundant. With the increase in SA treatment degree (50–100%), the hydroxyl, epoxy, and carboxylic acid groups gradually decrease, and the sp^2^ carbon structure gradually recovers.

According to Figure 6c,d, the analysis of the reduced specimens of GO and different concentrations of sodium ascorbate (SA) is as follows: The peak of GO in the high binding energy region (286–289 eV) is relatively strong, indicating the presence of a large number of oxygen-containing functional groups, while the sp^2^ carbon peak is relatively weak. In the group with 10% SA addition, the peak corresponding to the oxygen-containing functional group (286–289 eV) began to weaken but still exhibited a significant residual intensity. Meanwhile, the intensity of the sp^2^ carbon peak (284.5 eV) slightly increased, indicating the partial reduction of the material. In the group with 50% SA added, the high binding energy peak was further weakened, and the peak areas of C-O and C=O decreased; the sp^2^ carbon peak was significantly enhanced, indicating an improvement in the degree of reduction and the gradual recovery of the conjugated structure of graphene. In the group with 100% SA addition, the peaks of oxygen-containing functional groups almost disappeared (the binding energy 286–289 eV signal is extremely low); the sp^2^ carbon peak (284.5 eV) became the dominant peak, indicating a high degree of reduction and approaching the electronic structure of the original graphene. With the increase in SA concentration, the main peak position gradually shifted left from ~286 eV (GO) to 284.5 eV (100% SA), indicating that the chemical environment of carbon changed from the oxidation state (high binding energy) to sp^2^ carbon (low binding energy). The C1s peak of GO is relatively wide (with multiple components superimposed), while the peak shape becomes sharper after reduction, reflecting an improvement in chemical homogeneity.

**Figure 7:**
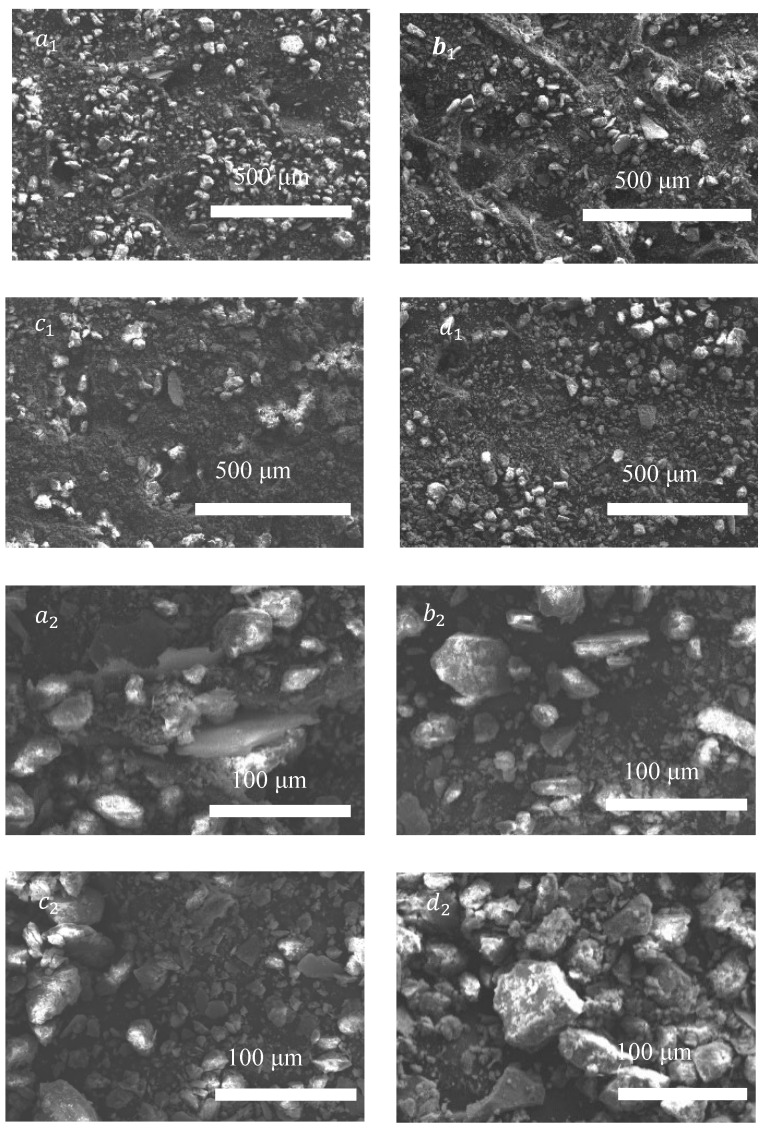
Electron microscopic image of the oxidation gradient of GO, (**a_1_**,**a_2_**) unreduced GO (0% SA) at 500 μm and 100 μm, (**b_1_**,**b_2_**) 10% SA–reduced GO at 500 μm and 100 μm, (**c_1_**,**c_2_**) 50% SA–reduced GO at 500 μm and 100 μm, and (**d_1_**,**d_2_**) 100% SA–reduced GO at 500 μm and 100 μm.

All SEM images revealed no distinct morphological differences between pristine soil and GO-composite soils. Figure 7 shows the electron microscope images of different oxidation gradients of GO. It can be seen that Figure 7a_1_–d_1_ correspond to unreduced GO, 10% SA–reduced GO, 50% SA–reduced GO, and 100% SA–reduced GO at 500 micrometers, while Figure 7a_2_–d_2_ correspond to the same oxidation gradients at 100 micrometers. This similarity may arise from (i) the homogeneous dispersion of GO sheets within the soil matrix, (ii) the sub-resolution scale of GO modifications relative to soil particle sizes, or (iii) potential masking effects of soil heterogeneity. Therefore, this result indirectly suggests that GO exhibits good compatibility with soil and maintains effective dispersion, resulting in no observable aggregation phenomena.

**Figure 8:**
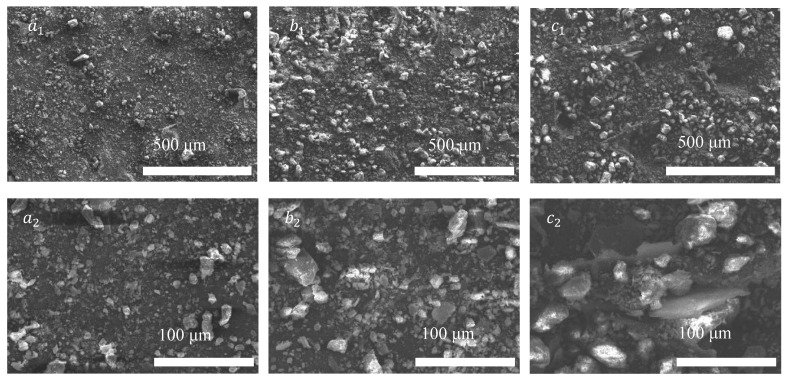
Electron microscopic image of the content gradient of GO, (**a_1_**,**a_2_**) the content is 0.005% GO at 500 μm and 100 μm, (**b_1_**,**b_2_**) the content is 0.01% GO at 500 μm and 100 μm and (**c_1_**,**c_2_**) the content is 0.03% GO at 500 μm and 100 μm.

According to the electron microscope image in Figure 8, which shows the different contents of GO. Figure 8(a_1_,b_1_,c_1_) corresponds to contents of 0.005% GO, 0.01% GO, and 0.03% GO at 500 micrometers, while Figure 8(a_2_,b_2_,c_2_) corresponds to the same contents at 100 micrometers. The sample with a GO content of 0.005% shows fewer GO particles in Figure 8(a_1_,a_2_), and the distribution between the particles is relatively uniform; Figure 8(b_1_,b_2_) shows that, in the sample with a GO content of 0.01%, the number of GO particles has increased and certain agglomeration phenomena have begun to occur. The improvement effect of GO is gradually emerging and has a positive impact on the water-holding capacity and stability of the soil. Figure 8(c_1_,c_2_) shows that in the sample with a GO content of 0.03%, the distribution of GO particles is denser and the agglomeration phenomenon is obvious. At this point, the introduction of GO significantly improved the microstructure of the soil, enhancing its water-holding capacity and erosion resistance. The optimal dosage of GO was found to be 0.03%.

**Figure 9:**
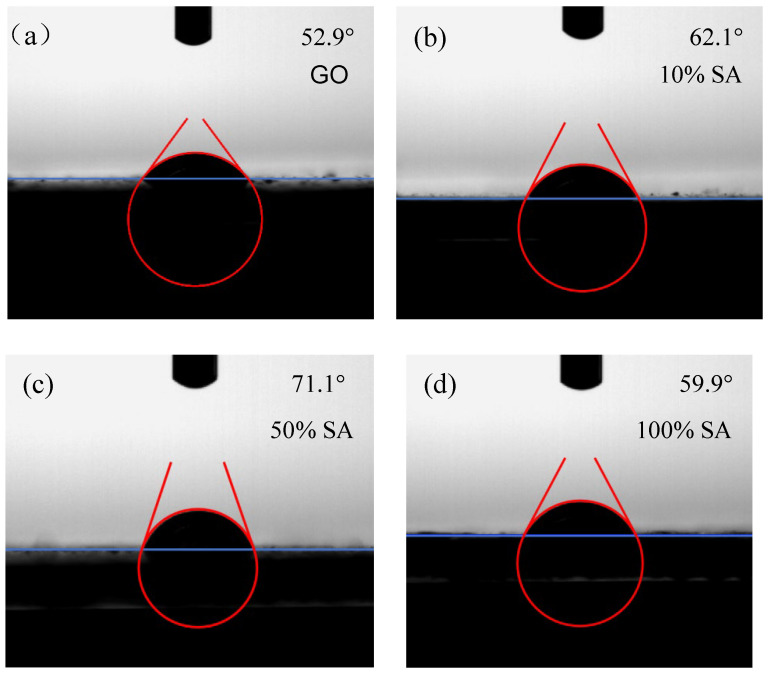
The contact angle of the oxidation gradient of GO, (**a**) 0% SA, (**b**) 10% SA, (**c**) 50% SA, and (**d**) 100% SA.

To evaluate the wetting performance, contact angle measurements were conducted on GO specimens with varying degrees of oxidation. Figure 9 shows the contact angles of GO with different oxidation levels. It can be seen that the addition of SA increases the contact angle of the base solution, causing the contact angle of GO to show a trend of first increasing and then decreasing. The contact angle of 0% SA is the smallest, at 52.9°. The contact angle of 50% SA is the largest, at 71.1°. As the amount of SA increases, GO is completely reduced, and the contact angle decreases to 59.9°. Based on the SEM images shown in Figure 8, it can be seen that the particles of 50% SA are relatively small and uniform and the contact angle is relatively larger. This suggests the successful hydrophobic modification of the soil surface, a property that could potentially benefit arid-land agriculture by (i) delaying drought stress through slower water evaporation and (ii) preventing fertilizer loss via controlled infiltration rates [28]. It can be determined that 50% is the optimal oxidation degree.

## 4. Conclusions

In this work, the influence of GO surface structure on the efficacy of loess soil remediation was systematically investigated via controlling the oxidation degree of GO by SA. It was found that adding 0.03% GO significantly reduced the disintegration amount of loess, indicating that the introduction of a higher dosage of GO made the combination between soil particles tighter, thereby forming a more stable soil structure. Moreover, with the increase in GO reduction degree, its influence on soil moisture retention capacity presents a certain pattern. A moderate reduction degree (50%) of GO can optimize the pore distribution of the soil and enhance its water retention capacity, while excessive reduction (100%) leads to a decrease in water retention capacity. This is because the interlayer stacking of high-reducibility GO is denser and its hydrophobicity is enhanced, resulting in a decrease in its dispersibility in the soil and thus affecting its combination with soil particles. Subsequently, long-term field trials can also be conducted on the basis of this research to evaluate the persistence and effect of GO in practical applications or explore the combined effect of GO with other soil improvement materials, with the aim of achieving better soil improvement results. In conclusion, through systematic experiments and analyses, this study has revealed the application potential of GO in the improvement of loess soil, providing an important theoretical basis and practical guidance for future soil remediation and ecological restoration.

## Data Availability

The raw data supporting the conclusions of this article will be made available by the authors on request.
